# Dysplastic ganglion cell tumor of the right cerebellum: A case report and literature review

**DOI:** 10.1097/MD.0000000000040990

**Published:** 2024-12-13

**Authors:** Shilong Wang, Jun Li, Jiangtao Dong, Ganggang Wang, Haoxiang Xu, Licang Zhu, Hui Xu

**Affiliations:** a Department of Neurosurgery, The First Affiliated Hospital of Shihezi University, Shihezi, China; b Department of Dermatology, The First Affiliated Hospital of Shihezi University, Shihezi, China.

**Keywords:** case report, dysplastic ganglion cell tumor, Lhermitte–Duclos disease, review

## Abstract

**Rationale:**

This study aims to present a case of cerebellar dysplastic ganglioneuroma, which is commonly referred to as Lhermitte–Duclos disease (LDD). Furthermore, the study aims to provide an extensive review of the essential aspects of LDD, thereby providing essential information for its accurate diagnosis and effective treatment.

**Patient concerns:**

A 54-year-old woman was admitted with symptoms of headache, facial numbness, and a visible cerebellar mass. Imaging studies revealed specific features such as the “tiger stripe sign” on magnetic resonance imaging, including hydrocephalus compression and abnormal vasculature.

**Diagnoses:**

The diagnosis of LDD was made.

**Interventions:**

The cerebellar mass was resected via a paracentral approach.

**Outcomes:**

The patient underwent surgery for a cerebellar dysplastic ganglion cell tumor (WHO grade I), confirmed by postoperative pathology. Despite sub-complete resection with minor residuals, the patient experienced significant improvement in symptoms. A postoperative computed tomography scan revealed a large cavity with frontal lobe hemorrhage. PTEN gene testing was recommended but declined due to financial constraints. The patient was discharged without complications.

**Lessons:**

LDD presents both benign and tumor characteristics, with a low likelihood of malignancy. Total resection is the recommended treatment, although challenges in complete excision may lead to recurrence. The importance of considering Cowden syndrome and genetic testing, particularly the PTEN gene, in patients with LDD, is emphasized. Long-term follow-up care is crucial for monitoring recurrence and related conditions.

## 1. Introduction

Cerebellar dysplastic ganglioneuroma, also known as Lhermitte–Duclos disease (LDD), is a rare intracranial space-occupying lesion primarily found in the cerebellum. It has a slow progression. In 2007, the World Health Organization classified it as a neuroepithelial tissue neoplasm consisting of neuronal and mixed neuronal glial tumors, grade I, with characteristics of misshapen and true tumors.^[1]^ According to the 2015 edition of Modern Neurosurgery, only 71 cases have been reported in the literature, and sporadic reports have emerged in recent years, totaling nearly 300 reported cases.^[2]^ It is still classified as a rare tumor. However, many clinicians lack knowledge of this disease due to insufficient clinical data. We recently admitted a patient with suspected LDD, which was subsequently confirmed by post-surgical pathology. In this report, we present the details of this case.

## 2. Case report

The patient, a 54-year-old woman, was admitted to the hospital on July 2, 2023, with an intermittent headache for over 6 months and numbness on the left side of her face for >2 weeks. She had been experiencing goiter for 24 years and had a thyroidectomy in 2008. Following the surgery, she had been taking 150ugqdpo of oral eugenol for an extended time, but the goiter reappeared on the left side. During the physical examination, 4 measurements were found to be normal. The patient’s mental status was clear, but her spirit was low. She exhibited sensory loss on the left side of her face. Limb movement was still possible, but the finger-nose test on the right side was positive, indicating some coordination difficulties. The test for alternation was also positive. Moreover, the patient showed signs of difficulty in standing with her eyes closed. In the left anterior cervical region, a visible soft mass was observed, measuring approximately 5 × 5 cm. Additionally, a tough subcutaneous mass with a clear boundary was found on the right forearm.

**Figure 1.**
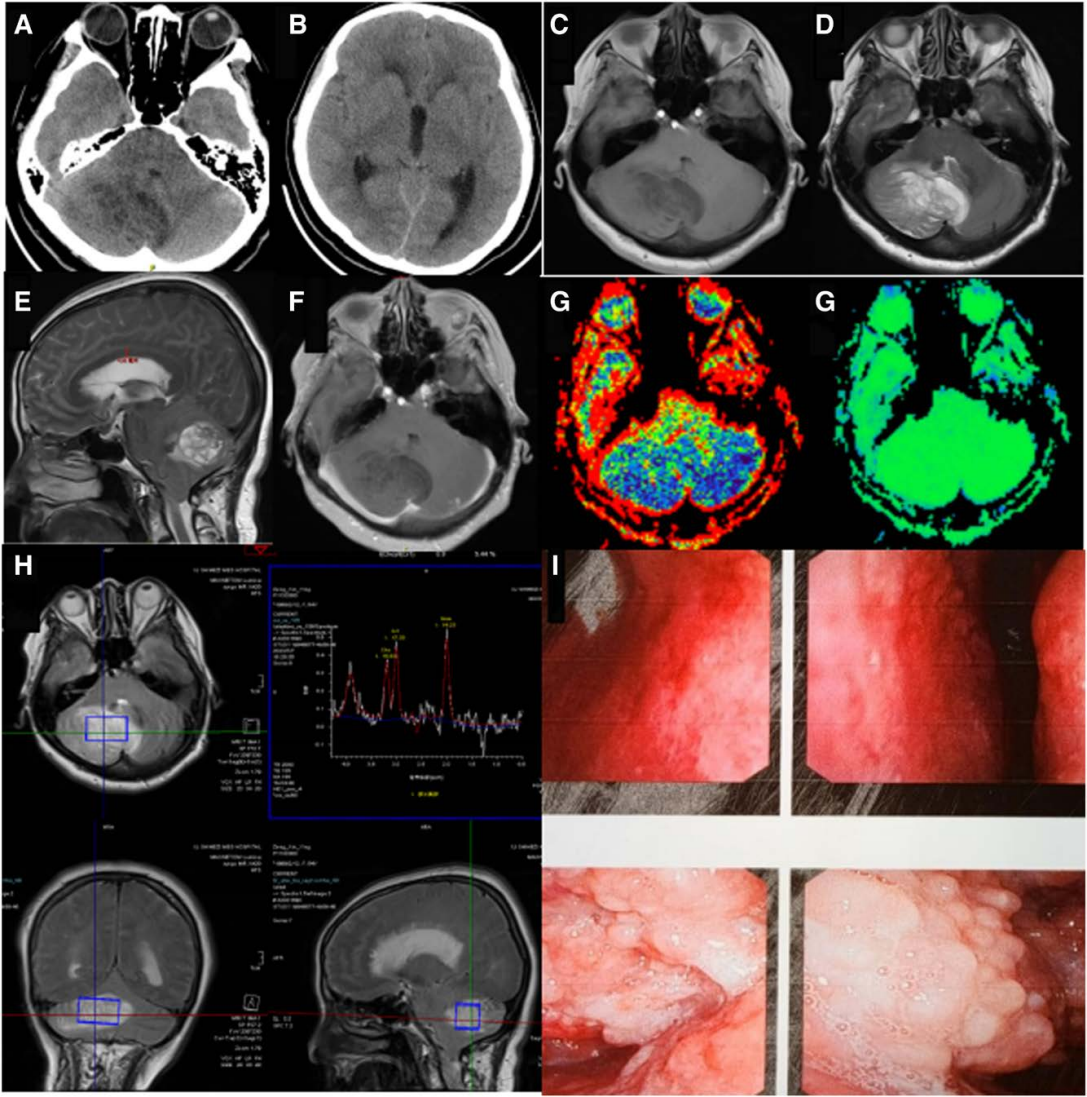
The typical imaging findings of Lhermitte–Duclos disease and the performance of laryngoscopy. The lesion exhibits striped iso-hyperdensity on unenhanced CT (A), shows low intensity on T1-weighted imaging (C), iso-hyperintensity on T2-weighted imaging (D), and reveals a tiger-striped lesion in the right cerebellar hemisphere (F). There was no apparent enhancement on contrast-enhanced T1-weighted imaging. (A, B, and E) The involvement of the corpus callosum, cerebellar vermis, and the posterior part of the pons is observed, with compression of the third and fourth ventricles. Perfusion-weighted imaging (G) and magnetic resonance spectroscopy (H) were also performed. Laryngoscopy indicates a wart-like fatty organism (I). CT = computed tomography.

The computed tomography (CT) scan revealed a patchy low-density shadow in the right cerebellum with non-uniform internal density. The larger cross-section of the shadow measured approximately 59 mm × 34 mm, suggesting the presence of a tumor. Enhanced magnetic resonance imaging (MRI) was then performed. The compression of the fourth ventricle caused obstructive hydrocephalus (Fig. 1A, B). The enhanced MRI scan showed a strip-like alternating distribution shadow with low signal intensity and iso-signal intensity in TIWI (Fig. 1C). In T2WI, there was a strip-like alternating distribution shadow with iso-signal intensity and high signal intensity, displaying a clear border. However, the border was not depicted in the anterior edge, which appeared as a band-like slightly high signal shadow in T2WI (Fig. 1D). The larger cross-section measured approximately 72 mm × 45 mm. Furthermore, the compression of the fourth ventricle resulted in mild hydrocephalus in the third ventricle and the lateral ventricles on both sides. The compression also caused thinning of the corpus callosum (Fig. 1E). The enhancement scan did not show obvious enhancement (Fig. 1F). Perfusion-weighted imaging revealed an abnormal perfusion signal area with a blurred edge in the right cerebellum (Fig. 1G). Magnetic resonance spectroscopy indicated that the N-acetyl aspartate/creatine (Cr) ratio was 14.23/17.33 and the choline/Cr ratio was 15.53/17.33. Both ratios were lower compared to the contralateral normal side. Additionally, there were lactate peaks observed in the abnormal area (Fig. 1H). Cranial MRI identified focal abnormal venous vascular aggregates in the right superior frontal gyrus, indicative of a small venous tumor.

The patient presented with follicular hyperplasia of the mucosa in both tonsils and the root of the tongue, accompanied by multiple local ulcers (Fig. 1I). The pathology findings revealed the following: microscopically, the lesion at the root of the tongue appeared to be a cyst with chronic inflammatory infection of the cystic wall and surrounding mucosa. Additionally, some areas showed vesiculitis and reactive hyperplasia of lymphoid follicles. Furthermore, a cyst in the nasopharyngeal area with partial squamous epithelium was observed. Immunohistochemistry tests showed partial positivity for CD3, partial positivity for CD20, positive staining for AE1/3 (epithelial), and positive staining for CD21 (follicular). Thyroid ultrasound indicated diffuse lesions along with multiple hypoechoic sheets and a slightly hyperechoic nodule located in the middle of the left lobe. According to the TI-RADS-US classification, these findings were categorized as category 3.

**Figure 2.**
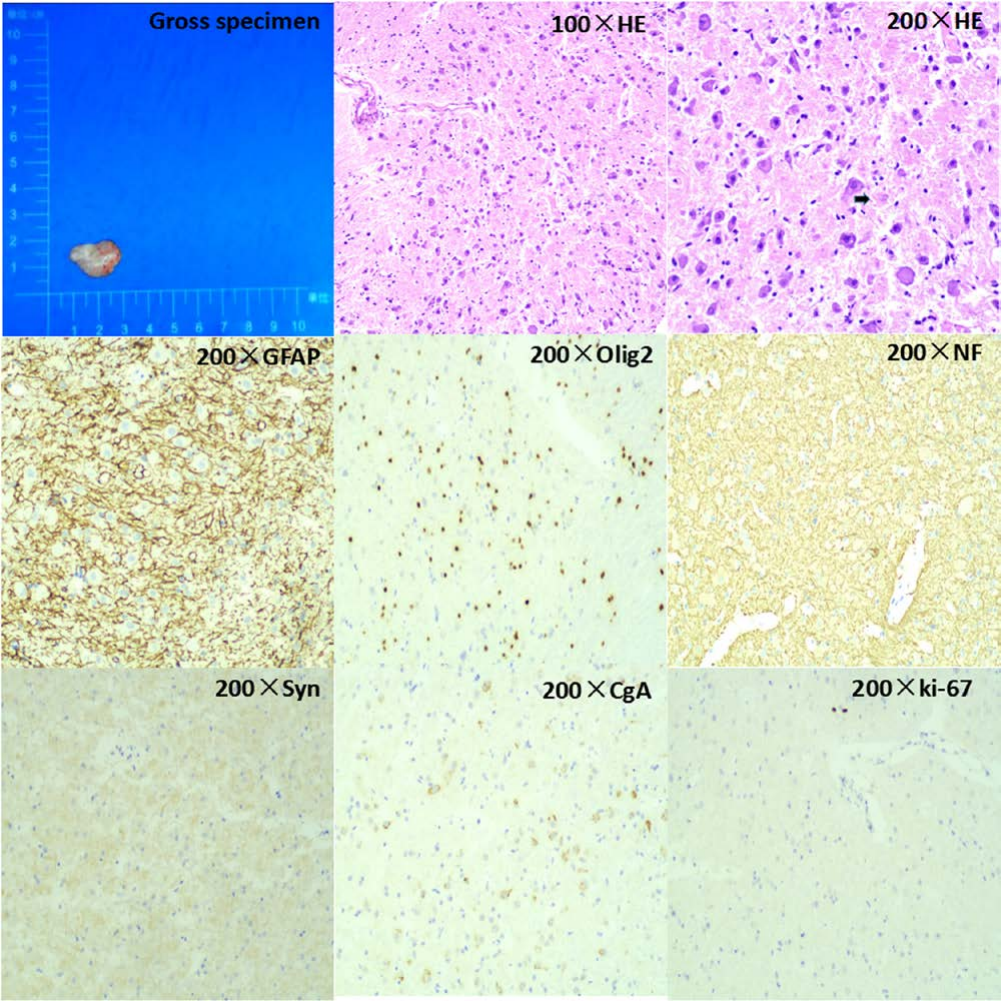
HE staining and immunohistochemical staining images.

The cerebellar mass was resected on July 10, 2023, via a paracentral approach. The intraoperative frozen biopsy indicated a benign tumor with characteristics of a cerebellar dysplastic ganglion cell tumor. The postoperative pathological analysis with paraffin sections confirmed the presence of a cerebellar dysplastic ganglion cell tumor, classified as World Health Organization grade I. Immunohistochemistry findings revealed positive expression of GFAP, scattered Olig2, NF, Syn, cgA, and occasional ki-67 (Fig. 2).

**Figure 3.**
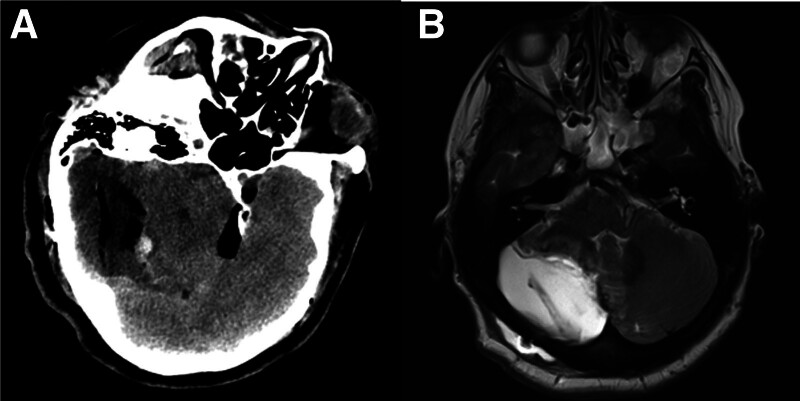
Postoperative CT (A) and MRI (B). CT = computed tomography, MRI = magnetic resonance imaging.

The postoperative CT scan displayed a large cavity in the tumor area along with a minor amount of hemorrhage in the frontal lobe, specifically, the region where the cavernous hemangioma was identified (Fig. 3A). The tumor was found to be sub-completely resected, with a small number of residuals on the postoperative T2WI of the MRI (Fig. 3B). Despite this, the patient experienced significant improvement in postoperative headache, symptoms of facial hyperalgesia, and ataxia, leading to the decision to discharge the patient from the hospital after the successful healing of the incision, free from any complications. In the postoperative period, the patient was advised to undergo further testing of the PTEN gene, but due to financial reasons, the family declined to proceed with this additional testing.

## 3. Discussion

### 3.1. Pathogenesis

Lhermitte and Duclos first described and named LDD in 1920. It is a rare cerebellar lesion with an unknown etiology and pathogenesis.^[3]^ Due to the different viewpoints on its pathogenesis, some researchers have referred to LDD as a Purkinje cell tumor, misshapen and dysplastic ganglion cell tumor, cerebellar developmental malformation, or cerebellar tumor. However, current evidence predominantly indicates that LDD is a misshapen or benign tumor, as no cases of malignancy have been reported. Support for the tumor claim is limited to cases of sub-total resection and recurrence. Some scholars suggest a potential correlation between LDD and Cowden syndrome, although the pathogenesis remains uncertain.^[4]^ Cowden syndrome is an uncommon autosomal dominant genetic syndrome, characterized by the presence of multiple cutaneous and mucosal misshapen tumors that tend to undergo malignant transformation. These malignant tumors are more prevalent in the breast, thyroid, and reproductive and digestive systems. LDD patients may present with various co-occurring malformations, including megalencephaly, spinal cord cavernous disease, polydactyly, and neurofibromatosis. Several genes, such as EGRF, SDHB-D, PIK3CA/AKT1, and PTEN, have been linked to Cowden syndrome. The PTEN gene is considered the primary causative gene for this syndrome, with mutations detected in numerous LDD patients.^[5–8]^

### 3.2. Clinical features

Our patient presented a rare case of LDD occurring in the right cerebellar hemisphere, whereas LDD mainly occurs in the left cerebellar hemisphere with less frequent invasion of the contralateral cerebellum. In this report, the LDD in our patient occurred in the right cerebellar hemisphere, which is one of the rare cases of LDD. LDD does not show a male–female predisposition in the population^[9]^ and commonly affects individuals between 30 and 40 years old.^[10]^ The youngest recorded case is 3 years old, while the oldest is 75 years old.^[11]^ LDD is often asymptomatic and typically detected through diagnostic imaging. When symptomatic, the clinical presentation varies and commonly includes headache, and cerebellar-related symptoms such as ataxia, hydrocephalus, and cerebral nerve palsy. These symptoms are primarily associated with increased intracranial pressure caused by the occupying effects of the lesion, impaired cerebellar function, obstruction of cerebrospinal fluid circulation, and compression of cerebral nerves.^[12]^ In some cases, symptoms may also include diplopia, psychiatric disorders, swallowing disorders, walking disorders, and other acute onset symptoms.^[13]^ The case in our report exhibited headache, ataxia, and cerebral nerve palsy, which align with the aforementioned common symptoms. Numerous studies have indicated a frequent association between LDD and Cowden syndrome. Therefore, it is important to be vigilant for the presence of tumors and malformations in various locations, including the skin, thyroid, breast, gastrointestinal tract, spinal cord, and spine. In the present case, the patient had a history of thyroidectomy. However, the patient and her family members were unable to provide clear information regarding whether the tumor was detected through pathological testing after the previous surgery. Additionally, there was a lack of medical examination records for the patient. Nevertheless, the patient exhibited an enlarged neck and a thyroid ultrasound suggesting a solid nodule. Subcutaneous swelling was also observed on the right forearm. We performed a cervical spine MRI, which did not reveal any spinal cord lesions such as spinal cord cavitation. Based on the patient’s clinical manifestations, we hypothesized that she had a combination of Cowden syndrome, which is characterized by a familial genetic predisposition. Further testing of the PTEN gene could have been conducted, but the patient declined. Additionally, there was no evidence of the relevant disease in the patient’s family history. Despite the patient’s refusal to pursue genetic testing and no history of related diseases among relatives, it is advisable to schedule a follow-up appointment at a later stage.

### 3.3. Imaging features

The cranial CT scan revealed an isodense or hypodense shadow in the cerebellum, occasional calcification, and insignificant occupying effect. No enhancement was observed. The primary imaging tool for diagnosing LDD is cranial scanning with enhanced MRI. The most typical feature of LDD, referred to as the “tiger stripe sign,” is characterized by long T1 and long T2 signals on MRI, occasionally interspersed with isotropic T1 and isotropic T2 signals. These signals exhibit a low signal on T1WI and a high signal on T2WI. The cerebrospinal fluid in the cerebellar sulcus displays a low signal on T1WI and a high signal on T2WI, presenting a striated or layered appearance reminiscent of tiger stripes.^[14]^ This finding suggests the thickening of the cerebellar cortex with an abnormal layered structure. Moreover, peritumoral edema serves as an important diagnostic indicator, indicating no significant disruption of the blood-brain barrier or extracellular edema.^[15]^ Imaging changes such as intratumor hemorrhage, intratumor calcification, abnormal blood vessels, and inhomogeneous enhancement are considered atypical. In instances of late tumor detection and large tumor size, obstructive hydrocephalus may occur, as demonstrated in this case. The compression of the fourth ventricle and mild dilatation of the third ventricle and lateral ventricles were observed in this case, leading to chronic symptoms of increased intracranial pressure and neurological dysfunction. Perfusion-weighted imaging hyperperfusion is a crucial diagnostic indicator for LDD. However, it does not necessarily indicate active cellular proliferation, as it could be attributed to the proliferation of thin-walled venous blood vessels with enlarged lumens. SWI examination confirms the presence of abnormal vasculature surrounding the lesion, rather than implying a disruption of the blood-brain barrier. In the magnetic resonance spectroscopy examination, different measurements hold different significance. The reduced N-acetyl aspartate/Cr value in this patient may be caused by an increased proportion of abnormal neurons within the lesion and a relative decrease in the proportion of normal neurons. The decrease in choline/Cr value suggests that cell proliferation is not evident, leading to the possibility of a non-tumor lesion. The presence of a lactate peak indicates an increased glycolysis process rather than necrosis or malignancy.^[16–18]^ The decrease in the bile peak signifies cell degradation, while the decrease in the choline peak suggests the destruction of the blood-brain barrier. The imaging findings in this patient aligned with the previously mentioned studies.

### 3.4. Pathologic features

The final diagnosis of the disease still relies on pathological examination. LDD is characterized by abnormal thickening of the cerebellar lobes. In LDD, the granule cell layer and the Purkinje cell layer are histologically replaced by dysplastic neuronal cells, which lack noticeable anisotropy or karyorrhexis. A significant number of granule cell extensions can be observed in the molecular layer, and the granule layer is diffused with numerous abnormally large granule cells resembling Purkinje cells. This feature corresponds to the “tiger-stripe sign” on imaging. The lesions often exhibit microcysts and calcifications, along with a small population of glial cells and Rosenthal fibers.^[19]^ However, there is a lack of vascularity between the interstitial cells. Immunohistochemistry assays demonstrate that these specific ganglion cells express Purkinje cell synaptic proteins and surface membrane proteins, indicating a neuronal rather than glial origin of the tumor cells. Additionally, positive neuronal markers, including Syn, Nse, and Neun, were observed. The lack of significant proliferative activity in tumors is confirmed by the absence of Ki-67 expression.

### 3.5. Treatment

Considering the potential pathogenesis, clinical characteristics, and pathological findings of LDD, most experts believe that total excision of the lesion, while preserving normal brain tissue, is the optimal treatment for patients exhibiting clinical symptoms or demonstrating a large size of ventricular hydrocephalus and occupancy on imaging. The likelihood of recurrence in patients who undergo total excision is exceptionally low based on imaging. However, due to the unclear boundaries of the LDD lesion, achieving total excision can be challenging, and there have been occasional instances of recurrence following subtotal excision. Nonetheless, there have been no documented cases of distant metastasis or malignancy.^[20]^ Symptomatic patients with concurrent hydrocephalus may undergo laparotomy, while asymptomatic patients identified through physical examination often require surgical intervention during subsequent follow-ups.

## 4. Conclusion

In conclusion, LDD is a rare low-grade locoregional lesion primarily occurring in the cerebellar hemisphere. It exhibits both malignant and true tumor characteristics, and the diagnosis is typically confirmed through the presence of the characteristic “tiger stripe sign” on MRI and other examination results. Surgical resection currently represents the optimal treatment, with complete lesion removal being the preferred approach. Physicians should carefully assess the presence of Cowden’s syndrome and consider enhancing clinical examination and PTEN gene testing. Long-term regular follow-up is recommended for patients.

However, the study still has significant limitations. Firstly, the number of these diseases is small. Secondly, the long-term follow-up is not systematic, which makes it impossible to validly assess the effectiveness of current treatments. Moreover, most of the studies remain superficial, and there are very few studies at the cellular and genetic levels. Therefore, further research in this regard is needed.

## Author contributions

**Conceptualization:** Hui Xu.

**Data curation:** Shilong Wang, Jun Li.

**Formal analysis:** Shilong Wang, Jun Li.

**Funding acquisition:** Shilong Wang, Licang Zhu.

**Investigation:** Shilong Wang, Jun Li, Jiangtao Dong, Ganggang Wang.

**Project administration:** Haoxiang Xu, Hui Xu.

**Software:** Haoxiang Xu.

**Supervision:** Hui Xu.

**Validation:** Jiangtao Dong, Hui Xu.

**Writing – original draft:** Shilong Wang.

**Writing – review & editing:** Shilong Wang, Jun Li, Jiangtao Dong, Ganggang Wang, Haoxiang Xu, Licang Zhu, Hui Xu.
